# Prediction of Subsequent Vertebral Fracture After Acute Osteoporotic Fractures from Clinical and Paraspinal Muscle Features

**DOI:** 10.1007/s00223-024-01209-0

**Published:** 2024-05-07

**Authors:** Yuchao Xiong, Cici Zhang, Xiaopei Chen, Li Wu, Shaohua Liang, Ye Zhang, Junbing Huang, Wei Guo, Xuwen Zeng, Fan Xu

**Affiliations:** Department of Radiology, Guangzhou Red Cross Hospital (Guangzhou Red Cross Hospital of Jinan University), Guangzhou, China

**Keywords:** Osteoporosis, Compression fracture, Paraspinal muscles, Fat infiltration, Nomograms

## Abstract

To construct a nomogram based on clinical factors and paraspinal muscle features to predict vertebral fractures occurring after acute osteoporotic vertebral compression fracture (OVCF). We retrospectively enrolled 307 patients with acute OVCF between January 2013 and August 2022, and performed magnetic resonance imaging of the L3/4 and L4/5 intervertebral discs (IVDs) to estimate the cross-sectional area (CSA) and degree of fatty infiltration (FI) of the paraspinal muscles. We also collected clinical and radiographic data. We used univariable and multivariable Cox proportional hazards models to identify factors that should be included in the predictive nomogram. Post-OVCF vertebral fracture occurred within 3, 12, and 24 months in 33, 69, and 98 out of the 307 patients (10.8%, 22.5%, and 31.9%, respectively). Multivariate analysis revealed that this event was associated with percutaneous vertebroplasty treatment, higher FI at the L3/4 IVD levels of the psoas muscle, and lower relative CSA of functional muscle at the L4/5 IVD levels of the multifidus muscle. Area under the curve values for subsequent vertebral fracture at 3, 12, and 24 months were 0.711, 0.724, and 0.737, respectively, indicating remarkable accuracy of the nomogram. We developed a model for predicting post-OVCF vertebral fracture from diagnostic information about prescribed treatment, FI at the L3/4 IVD levels of the psoas muscle, and relative CSA of functional muscle at the L4/5 IVD levels of the multifidus muscle. This model could facilitate personalized predictions and preventive strategies.

**Supplementary Information:**

The online version contains supplementary material available at 10.1007/s00223-024-01209-0.

## Introduction

Osteoporosis represents a major public health concern and its importance is growing in the context of the aging global population [1]. One in every two women, and one in every five men, develop a fragility fracture at some point [2]. Osteoporotic vertebral compression fracture (OVCF) is a common and severe consequence of osteoporosis affecting an estimated 1.4 million people per year worldwide [3]. Patients who experience OVCF are at increased risk of subsequent fractures [4–6]: approximately 20% of OCVF patients develop new vertebral fractures within 1 year [7–9], and approximately 38% within 2 years [10]. This risk of subsequent fractures is greatest within 3 months of a fracture event [11]. The high risk of subsequent fractures during the first 2 years after a fracture is referred to as the imminent risk period [4]. Subsequent vertebral fractures can lead to increased kyphosis, severe deterioration of quality of life, severe functional impairment, and increased mortality [12]. The introduction of fracture liaison services may reduce patient mortality and the risk of subsequent vertebral fracture [13]; it is therefore important to identify risk factors for subsequent vertebral fracture within 3 months and 2 years of the initial fracture event, and to enhance measures and services to improve the prognosis of OVCF.

Many clinical and vertebral factors appear to increase the risk of subsequent vertebral fracture, including advanced age [14, 15], female sex [15], fractures in the thoracolumbar junction [15], high pain scores [16], low bone mineral density (BMD) [17], higher segmental Cobb angle[17], and low vitamin D levels [18]. However, the conclusions of previous studies have been inconsistent, and are only based on clinical and vertebral factors. The paraspinal muscles support and strengthen the spine, thus stabilizing its motion [19]. Impairment of the paraspinal muscles undermines spinal stability. Dysfunction of these muscles is associated with collapse of the fractured vertebra after OVCF [20]. The paraspinal muscles may also play an important role in subsequent vertebral fracture, although this was neglected in most previous studies.

The purpose of this study was to explore potential risk factors for recurrent vertebral fractures and assess the role of paraspinal muscles. Therefore, we developed and validated a nomogram based on clinical factors and paraspinal muscle features for predicting vertebral fractures occurring after acute OVCF.

## Materials and Methods

### Study Participants

This retrospective study was approved by the institutional review board of Guangzhou Red Cross Hospital (Ethics Approval Number: 2023-329-01, date of approval: 3 January 2023) and the need to obtain informed consent was waived. We reviewed picture archiving and communication system databases to recruit patients diagnosed with acute vertebral compression fracture (VCF) between January 1, 2013 and August 31, 2022. Acute VCF was diagnosed based on the presence of bone marrow oedema and vertebral compression on MRI. We applied the following inclusion criteria: (1) first acute single-segment VCF was at the level of T10–L5, (2) magnetic resonance imaging (MRI) diagnosis of acute VCF, (3) MRI included the L3/4 and L4/5 intervertebral discs (IVDs), (4) follow-up of at least 2 years, (5) availability of lumbar X-ray imaging at the time of initial diagnosis of VCF, and (6) availability of information about BMD based on dual energy X-ray absorptiometry. We adopted the following exclusion criteria: (1) evidence of a previous OVCF; (2) history of vertebral surgery, including vertebroplasty, spinal decompression, or fusion surgery; (3) presence of concurrent spinal tumor or pathological compression fracture; (4) presence of Parkinson’s disease, amyotrophic lateral sclerosis, or other neuromuscular disorders; (5) distraction or rotation injury associated with the vertebral fracture, (6) history of hip fracture and glucocorticoid use, and (7) incomplete follow-up data.

Patients’ data meeting the above criteria were retrospectively analyzed and they were divided into two groups depending on the occurrence (or lack thereof) of a vertebral fracture during follow-up: the subsequent vertebral fracture group (hereafter referred to as the “fracture” group) and non-subsequent vertebral fracture group (hereafter referred to as the “non-fracture” group). The diagnostic standard for establishing occurrence of subsequent vertebral fracture was the presence of a fresh VCF (presence of vertebral bone marrow edema with no occupying effect and no signs of infection) within adjacent and non-adjacent vertebrae on MRI.

### Imaging Procedures

MRI and radiographic images of the lumbar spine were obtained using a 1.5 T scanner and digital X-ray system, respectively. Detailed information on these devices and the parameters used during examinations are in Supplementary Material 1.

### Data Collection and Image Analysis

#### Clinical Information

We extracted potential risk factors for subsequent vertebral fracture from the medical records, including age, gender, BMD, fracture time, treatment options for OVCF, and history of anti-osteoporosis treatment. We classified treatment options for OVCF into two categories: percutaneous vertebroplasty (PVP) treatment and non-surgical treatment. PVP is described in detail in Supplementary Material 2. History of anti-osteoporotic treatment was defined as application of anti-osteoporotic medications, including zoledronic acid, denosumab, and teriparatide, and patients who did not apply these medications were those who did not receive anti-osteoporosis treatment. Anti-osteoporotic therapy includes pre-first fracture anti-osteoporotic therapy, post-first fracture anti-osteoporotic therapy, and pre- and post- first fracture anti-osteoporotic therapy.

#### Radiographic and MRI Image Analysis

Two musculoskeletal radiologists (C.Z. and Y.X.) with 6 and 3 years of experience in musculoskeletal radiology, respectively, independently reviewed all MRIs and radiographs. When there was disagreement, the two radiologists reached a consensus through discussion with a senior radiologist (X.Z.) with 25 years of experience in musculoskeletal radiology.

A lumbar plain radiograph was obtained from patients placed in the lateral standing position during first acute compression fracture. The following imaging parameters were evaluated from radiographs: lumbar lordosis, local kyphotic angle, and fracture location. Lumbar lordosis is defined as the angle between the segment aligned with the L1 upper endplate and the segment aligned with the L5 lower endplate. The local kyphotic angle was measured between vertebrae adjacent to the fracture vertebrae using Cobb’s method [21]. Fracture location was classified into two categories: thoracolumbar junction (T10–L2) and non-thoracolumbar junction (L3–L5).

**Fig. 1:**
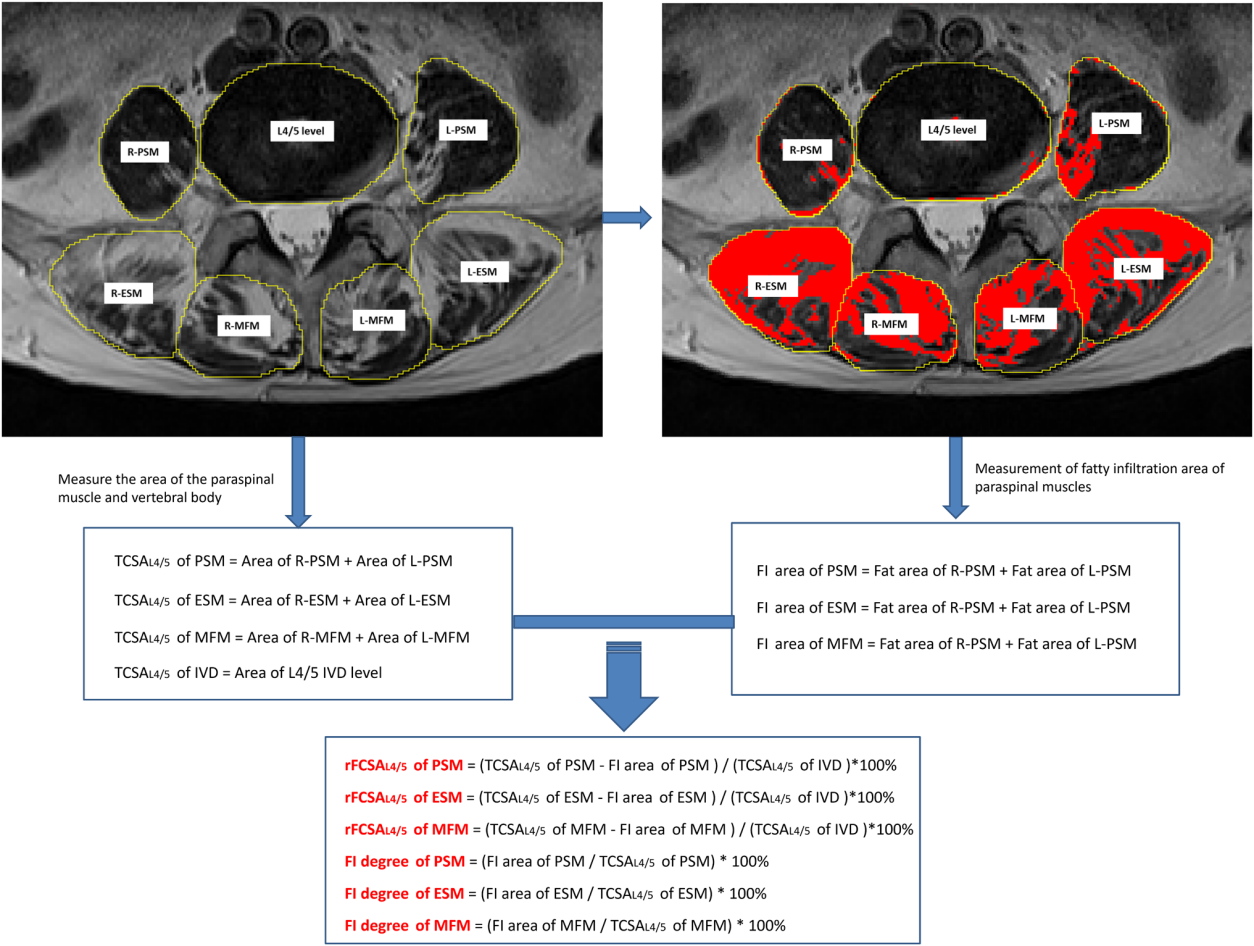
Measurement method and calculation formula of paraspinal muscles

We evaluated the following parameters from MRI images: cross-sectional area (CSA) of the paraspinal muscle, degree of fatty infiltration (FI) of the paraspinal muscles, compression fracture type, and subcutaneous fat thickness. We estimated CSA and FI using the open-source software ImageJ (version 1.53; National Institutes of Health, Bethesda, MD, USA). We estimated the CSA of the multifidus muscle (MFM), erector spinae muscle (ESM), and psoas muscle (PSM) at the level of the L3/4 and L4/5 IVDs, from the contours of the muscle fascia boundary. These contours were used to delineate regions of interest on T2-weighted images and the total selected muscle area was labeled “TCSA” (for total CSA). We automatically selected regions with different fat signal intensity using the “Moments dark” option of the auto-threshold method. The degree of muscle FI was defined as the ratio of fat area in the muscle to the total area of the muscle. Functional CSA refers to fat-free lean paraspinal muscle [20]. We also calculated the relative CSA (ratio between muscle CSA and CSA of the IVD on the corresponding level) to reduce the effect of body size on muscle parameters [22]. We labeled the relative CSA of total muscle (T) and functional muscle (F) as “rTCSA” and “rFCSA”, respectively. Figure 1 shows the measurement methods and calculation formulas adopted in this study. According to the OF classification [23], compression fractures are divided into five types: OF 1, vertebral body without deformation, only vertebral bone marrow edema apparent; OF 2, deformation, no or slight posterior wall (< 1/5) involvement; OF 3, significant posterior wall deformation (> 1/5); OF 4, loss of integrity of the vertebral framework or pincer fracture; and OF 5, distraction or rotation injury associated with the vertebral fracture. We defined subcutaneous fat thickness as the average thickness of the subcutaneous fat located on the lateral border of the bilateral ESM at the level of the L3/4 IVD.

Image analysis was performed by one of two trained musculoskeletal radiologists with 3 years of subspecialty experience (C.Z. and Y.X.). It takes approximately 2 min per patient to delineate the paraspinal muscles. We randomly selected 50 patients for repeated (2 ×) region of interest delineation, with an interval of 1 week between the two delineations to avoid recall bias.

### Statistical Analyses

Statistical analyses were performed using SPSS (version 22.0; IBM Corp., Armonk, NY, USA) and R software (version 4.2.1; R Foundation for Statistical Computing, Vienna, Austria). Inter-observer and intra-observer measurement reliability was assessed using single-measure intra-class correlation coefficients (ICC) with a two-way random model under absolute agreement. For continuous variables, the Kolmogorov–Smirnov test was used to assess normality, with *t* tests for those that followed a normal distribution and Mann–Whitney *U* tests for those that did not. For categorical variables, the Chi-square test and Fisher's exact test were used for comparison. We used univariate Cox analysis to assess the associations between different factors and subsequent vertebral fracture. We then submitted variables with *P* values < 0.1 in univariate analysis to multivariate analysis (forward stepwise Cox regression). We gradually excluded predictors until all predictors had a *P* value < 0.05, and defined the associated model as the final predictive model. The final risk factors were incorporated into R software to construct a nomogram prediction model. We used the area under the receiver operating characteristic curve (AUC) over time to assess the discriminatory ability of risk scores at 3 months, 12 months, and 24 months after vertebral fracture. We evaluated model prediction performance using the receiver operating characteristic curve (ROC), and assessed the prediction consistency and clinical utility of the nomogram using calibration curve and decision curve analyses (the predicted risk became closer to the standard curve, the conformity of the model improved). We assessed model accuracy and potential overfit via bootstrap internal validation with 1,000 resampling iterations, and calculated the optimism-corrected C-index.

## Results

### Patient Characteristics

**Fig. 2:**
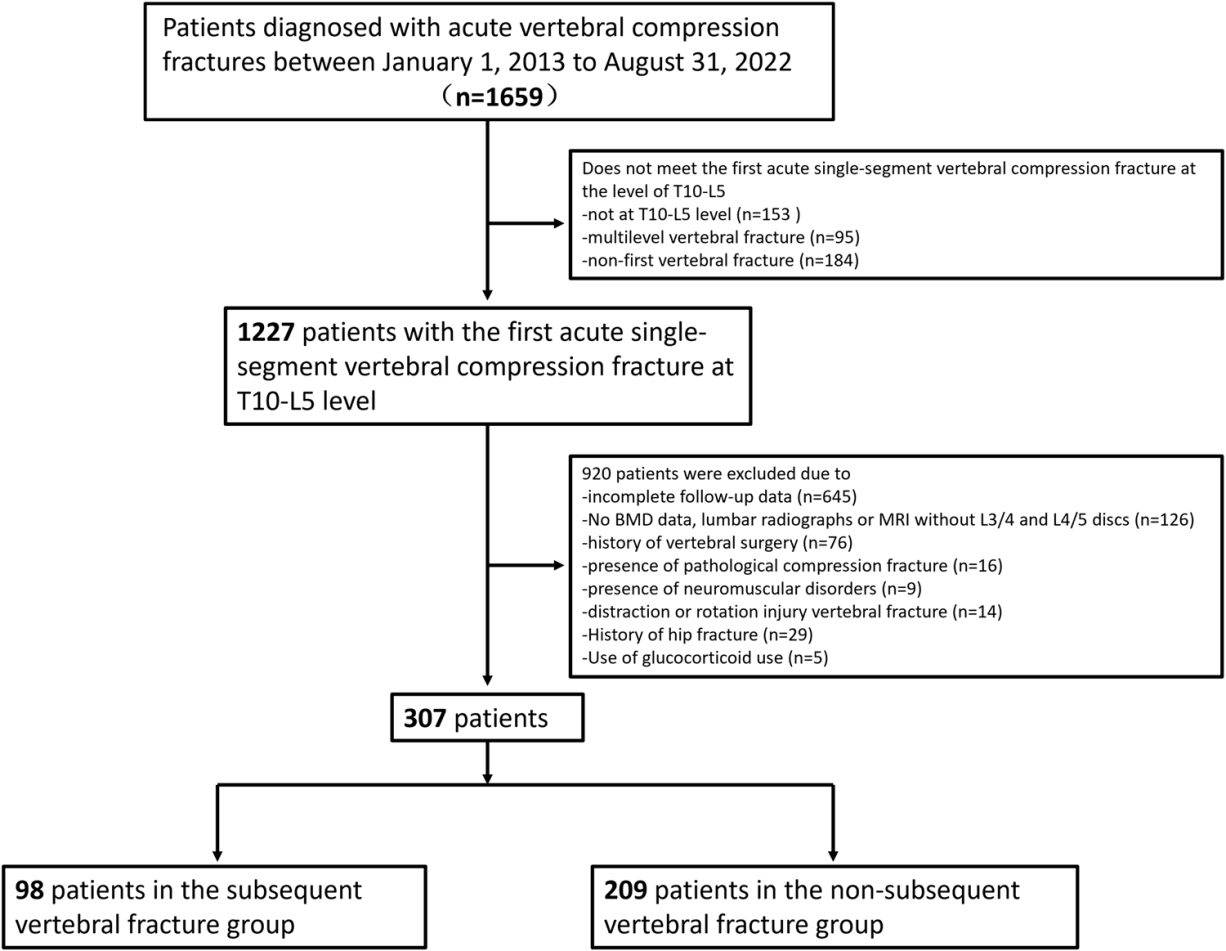
Flowchart of study enrollment

We initially included 1659 patients with acute compression fractures and further based on the inclusion and exclusion criteria, 307 patients were finally included and the detailed process is shown in the flowchart (Fig. 2). A total of 307 patients: 98 in the subsequent vertebral fracture group (median follow-up time: 6.40 months, range 0.23–23.77 months) and 209 in the non-subsequent vertebral fracture group (median follow-up time: 36.60 months, range 24.1–114.93 months). Subsequent vertebral fracture occurred within 3 months in 10.8% (33/307) of patients, 12 months in 22.5% (69/307) of patients, and 24 months in 31.9% (98/307) of patients. Anti-osteoporotic treatment was given to 66 patients, of whom 2 patients received treatment only before the first fracture (1 in the fracture group and 1 the in non-fracture group), 51 patients received treatment only after the first fracture (20 in the fracture group and 31 in the non-fracture group), and 13 patients received anti-osteoporosis treatment both before and after the first fracture (6 in the fracture group and 7 in the non-fracture group). Of the 66 patients, 53 were on zoledronic acid, 12 on teriparatide, and 1 on denosumab. Table 1 provides a summary of the patient characteristics.

**Table 1:** Patient characteristics of the study population

	Total (*n* = 307)	Non-fracture group (*n* = 209)	Fracture group (*n* = 98)	*P* value
Age (years)	78.39 ± 6.89	77.06 ± 9.05	81.22 ± 6.89	0.003
Gender				0.382
Male	63 (20.5%)	40 (19.1%)	23 (23.5%)	
Female	244 (79.5%)	169 (80.9%)	75 (76.5%)	
Fracture time				
Within 3 months	–	–	33	
Within 3 to 12 months	–	–	36	
Within 12 to 24 months	–	–	29	
Fracture location				0.572
Non-thoracolumbar junction	45 (14.7%)	29 (13.9%)	16 (16.3%)	
Thoracolumbar junction	262 (85.3%)	180 (86.1%)	82 (83.7%)	
Compression fracture type				0.341
OF 1	8 (2.6%)	5(2.4%)	3(3.1%)	
OF 2	105 (34.2%)	76 (36.4%)	29 (29.6%)	
OF 3	156 (50.8%)	104 (49.8%)	52 (53.1%)	
OF 4	38 (12.4%)	24 (11.5%)	14 (14.3%)	
Lumbar lordosis (°)	30.93 ± 12.38	30.85 ± 12.62	31.10 ± 11.89	0.375
Localkyphotic angle (°)	10.67 ± 8.48	10.33 ± 8.56	11.39 ± 8.31	0.688
BMD (g/cm^3^)	0.67 [0.63, 0.77] ± 0.13	0.69 [0.63, 0.78]	0.67 [0.62, 0.75]	0.044*
Subcutaneous fat thickness (mm)	30.47 ± 9.71	31.40 ± 9.31	28.49 ± 10.26	0.501
PVP treatment				0.000
No	90 (29.3%)	79 (37.8%)	11 (11.2%)	
Yes	217 (70.7%)	130 (62.2%)	89 (88.8%)	
Anti-osteoporosis treatment				0.077
No	241 (78.5%)	170 (81.3%)	71 (72.4%)	
Yes	66 (21.5%)	39 (18.7%)	27 (27.6%)	
Pre-first fracture				0.209
No	292 (95.1%)	201 (96.2%)	91 (92.9%)	
Yes	15 (4.9%)	8 (3.8%)	7 (7.1%)	
Post-first fracture				0.093
No	243 (79.2%)	172 (81.8%)	72 (73.5%)	
Yes	64 (20.8%)	38 (18.2%)	26 (26.5%)	
Pre- and post-first fracture				0.412
No	294 (95.8%)	202 (96.7%)	92 (93.9%)	
Yes	13 (4.2%)	7 (3.3%)	6 (6.1%)	

Quantitative data are reported as mean ± standard deviation or median with interquartile range; independent samples *t* tests were used (*Mann–Whitney *U* test was used). Qualitative variables are reported in raw numbers; percentages in parentheses, using Chi-square (*χ*^2^) tests or using Fisher’s exact test

*BMD* bone mineral density, *PVP* percutaneous vertebroplasty

### Inter- and Intra-observer Agreement and Measurement Reliability

Fourteen area parameters were manually outlined from lumbar spine MR images, that is, left paravertebral muscle area (L-PSM, L-ESM, and L-MFM), right paravertebral muscle area (L-PSM, L-ESM, and L-MFM), and IVD area at the L3/4 IVD level and L4/5 IVD level, respectively, with inter- and intra-observer ICCs greater than 0.90 for all the parameters. Results of the inter- and intra-observer agreement analyses are presented in Supplementary Material 3.

### Predictors of Subsequent Vertebral Fracture

The univariable Cox proportional hazard model revealed that the following factors were significantly associated with subsequent vertebral fracture: older age, PVP treatment, lower BMD, and lower rFCSA and higher FI of ESM, MFM, and PSM at the L3/4 and L4/5 IVD levels (Table 2).

**Table 2:** Univariate and multivariate Cox regression for subsequent vertebral fracture

Variable	Univariable analysis		Multivariable analysis	
	Hazard ratio	*P* value	Hazard ratio	*P* value
Age	1.53 (1.20–1.95)	0.001		
Gender	1.20 (0.75–1.92)	0.443		
Fracture location	0.87 (0.51–1.49)	0.61		
Compression fracture type	1.18 (0.84–1.65)	0.296		
Lumbar lordosis	1.03 (0.87–1.22)	0.741		
Local kyphotic angle	0.96 (0.82–1.11)	0.549		
BMD	1.26 (0.94–1.67)	0.117		
Subcutaneous fat thickness	0.80 (0.65–0.98)	0.034		
PVP treatment	3.99 (2.13–7.47)	0	2.95 (1.55–5.6)	0.001
Anti-osteoporosis treatment	0.86 (0.60–1.25)	0.442		
rFCSA_L3/4_ of ESM	1.75 (1.34–2.29)	0		
FI_L3/4_ of ESM	1.49 (1.15–1.92)	0.002		
rFCSA_L3/4_ of MFM	1.71 (1.28–2.29)	0		
FI_L3/4_ of MFM	1.59 (1.22–2.06)	0.001		
rFCSA_L3/4_ of PSM	1.46 (1.12–1.90)	0.005		
FI_L3/4_ of PSM	1.73 (1.35–2.21)	0	1.35 (1.04–1.75)	0.025
rFCSA_L4/5_ of ESM	1.62 (1.21–2.17)	0.001		
FI_L4/5_ of ESM	1.65 (1.25–2.18)	0		
rFCSA_L4/5_ of MFM	1.72 (1.39–2.13)	0	1.46 (1.16–1.84)	0.001
FI_L4/5_ of MFM	1.59 (1.25–2.04)	0		
rFCSA_L4/5_ of PSM	1.50 (1.20–1.88)	0		
FI_L4/5_ of PSM	1.54 (1.20–1.98)	0.001		

*BMD* bone mineral density, *PVP* percutaneous vertebroplasty, *rFCSA* relative functional cross-sectional area, *PSM* psoas muscle, *ESM* erector spine muscle, *MFM* multifidus muscle, *FI* fatty infiltration

Multivariable Cox proportional hazard analysis identified three independent predictors of the risk of subsequent vertebral fracture (Table 2): previous PVP treatment (hazard ratio [HR] 2.95; 95% confidence interval [CI] 1.55–5.60; *P* = 0.001), higher FI_L3/4_ of PSM (HR 1.35; 95% CI 1.04–1.75, *P* = 0.025), and lower rFCSA_L4/5_ of MFM (HR 1.46; 95% CI 1.46–1.84, *P* = 0.001).

### Development and Assessment of the Predictive Nomogram

**Fig. 3:**
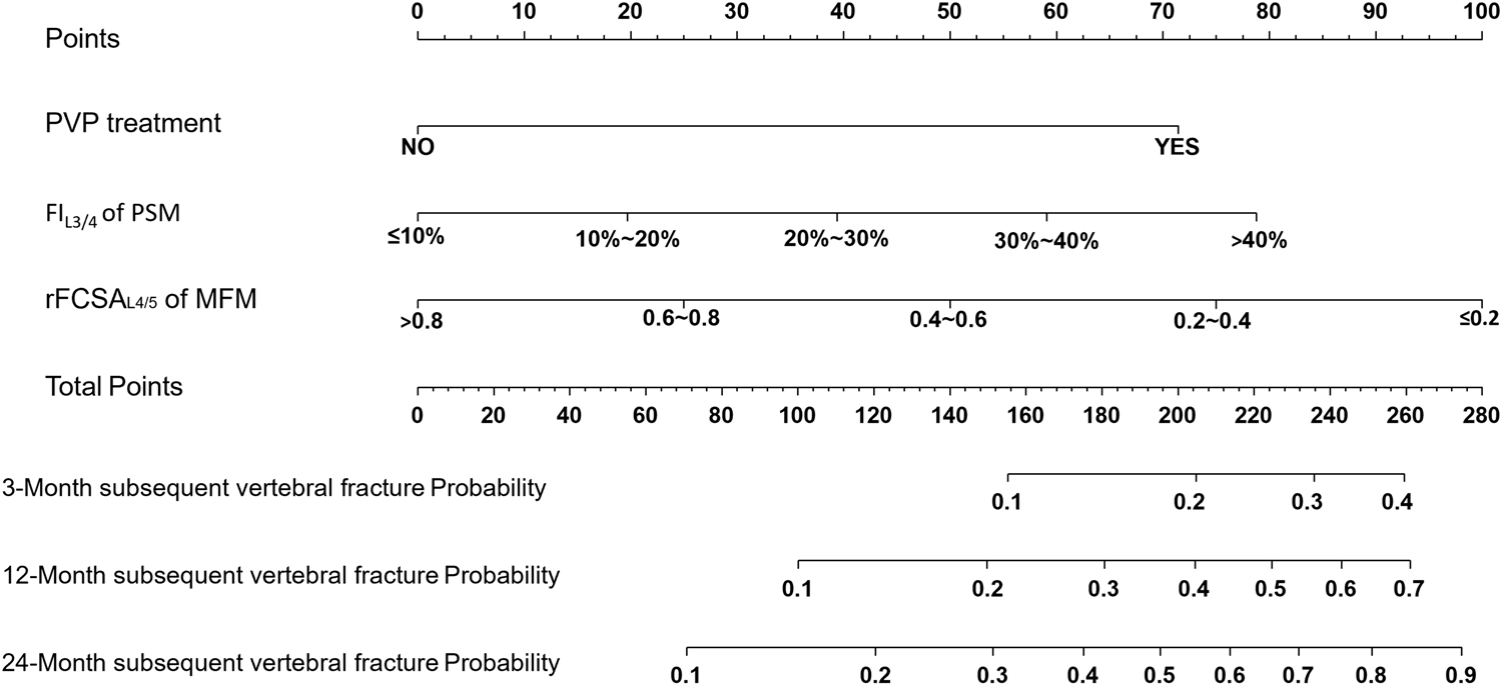
The nomogram to predict subsequent vertebral fracture was created based on three significant predictors

**Fig. 4:**
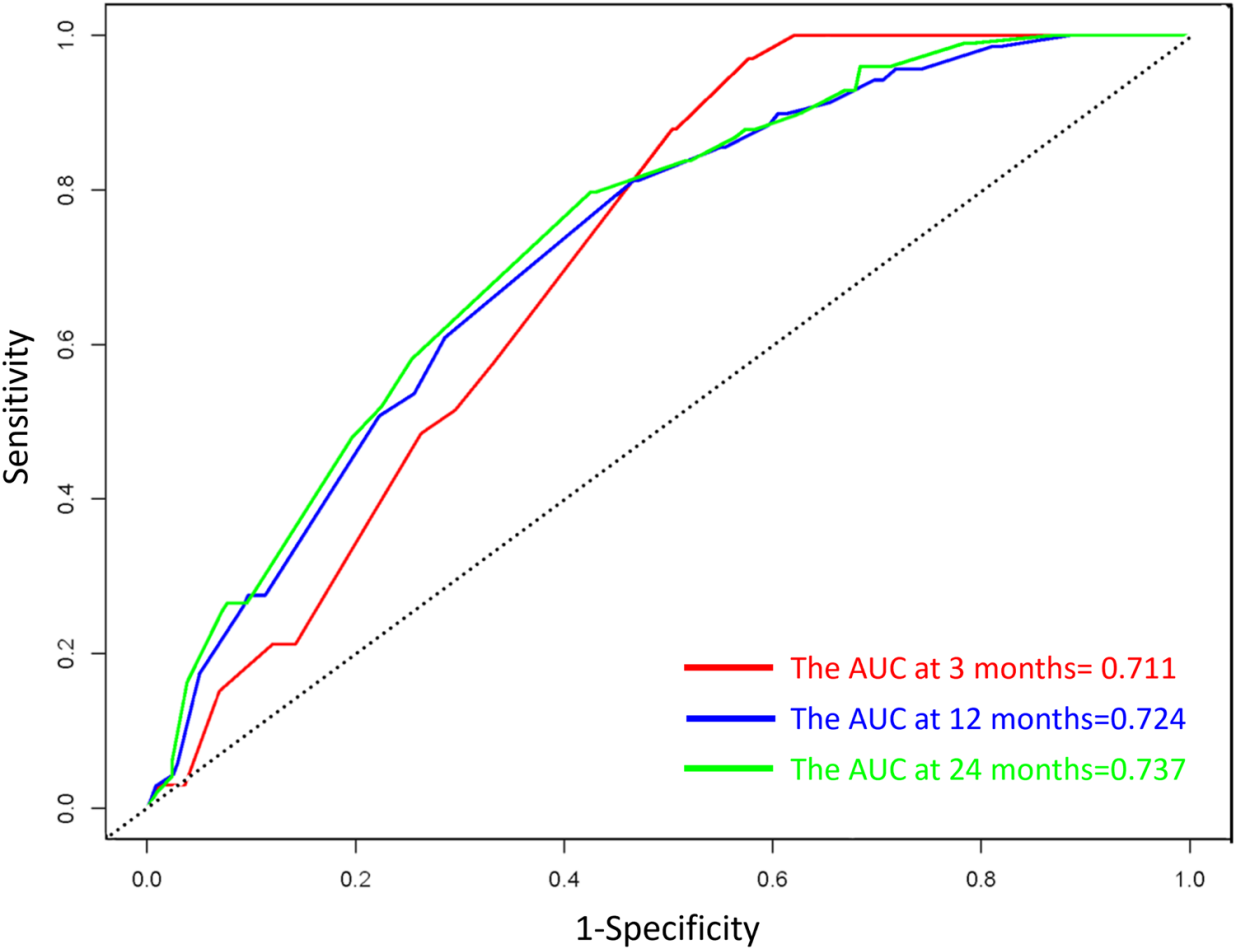
The time-dependent receiver operating characteristic (ROC) curve and area under the ROC curve (AUC)

**Fig. 5:**
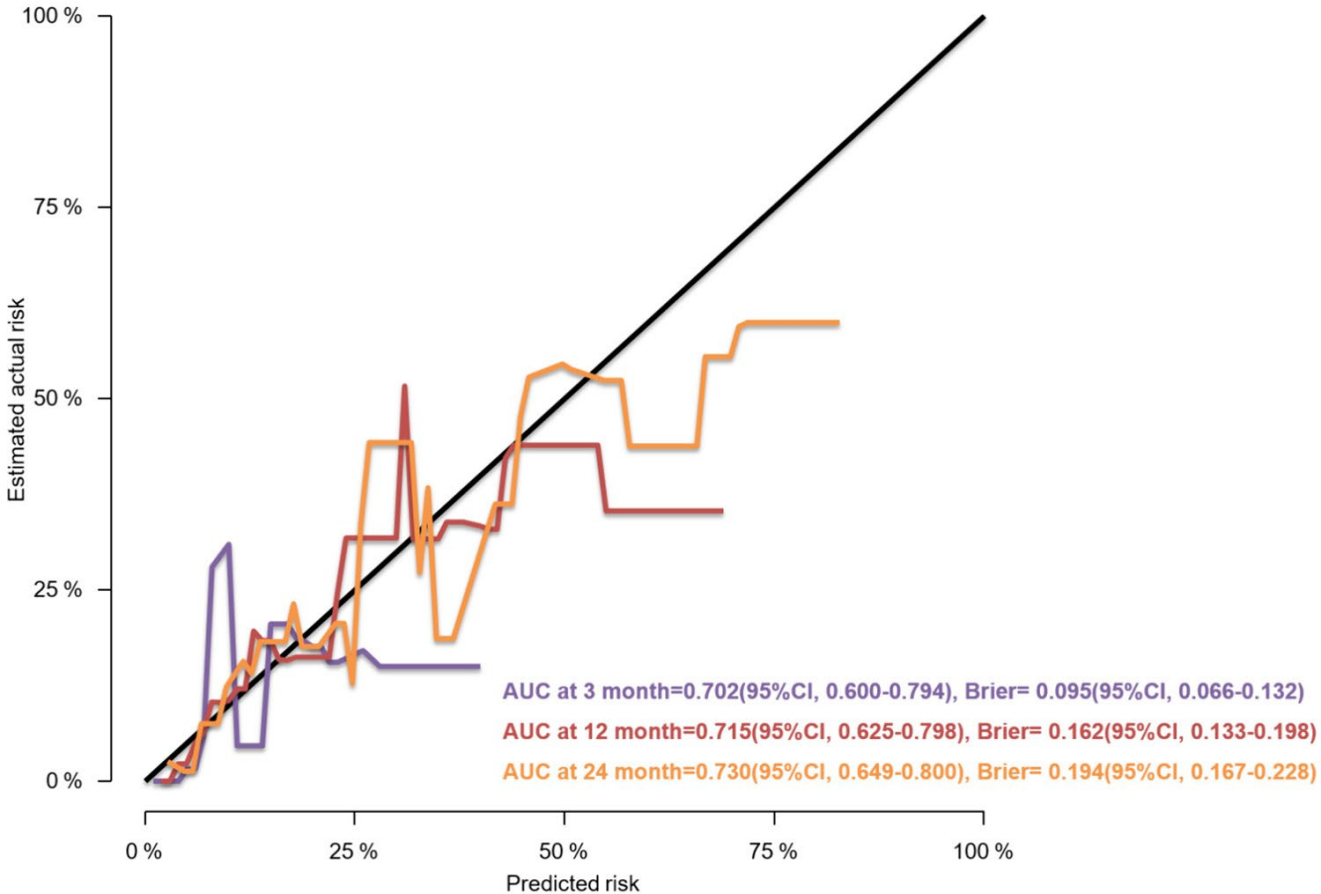
The calibration curves for evaluating the accuracy of the nomogram

**Fig. 6:**
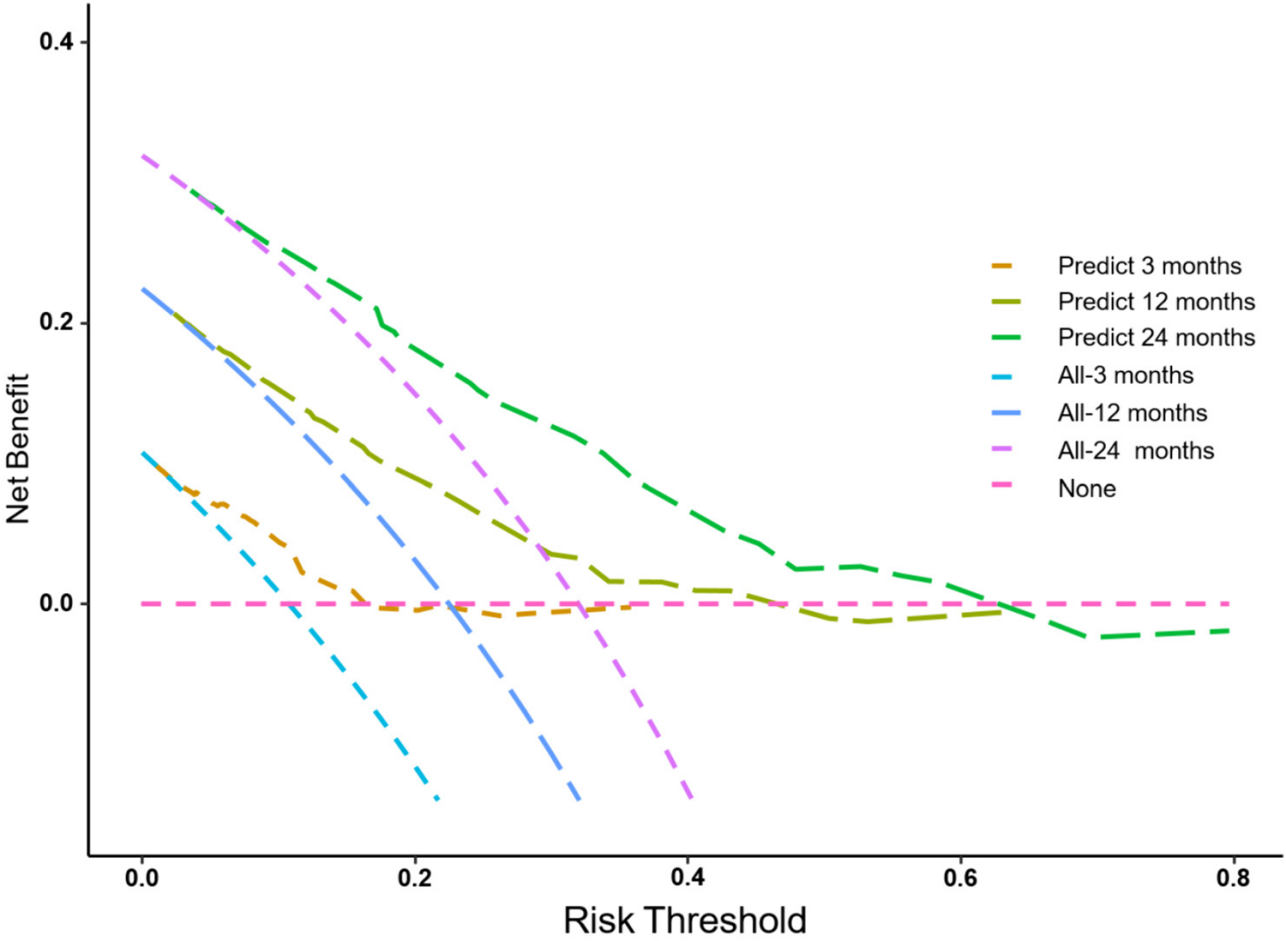
Decision curve analysis for the nomogram and the model with predictors

We used a stepwise Cox regression model to develop a nomogram for predicting subsequent vertebral fracture within 3, 12, and 24 months after OVCF with a C-index of 0.723 (95% CI 0.646–0.736). Higher total scores for predictors in the nomogram were associated with greater risk of subsequent vertebral fracture (Fig. 3). Internal validation confirmed good performance of the model. AUC values for predicting the risk of subsequent vertebral fracture were 0.711, 0.724, and 0.737 at 3, 12, and 24 months after OVCF, respectively (Fig. 4). After bootstrap validation with 1000 resampling iterations, the bias-corrected C-index values were 0.702, 0.714, and 0.730 at 3, 12, and 24 months after OVCF, respectively, indicating slight systematic overestimation by our model (Fig. 5). Decision curve analysis shows that the nomogram model has sufficient utility for real-world clinical applications (Fig. 6).

## Discussion

We developed and internally validated a nomogram based on clinical and paraspinal muscle characteristics to predict vertebral fractures subsequent to an acute osteoporotic vertebral fracture. The nomogram incorporates three factors: PVP treatment, degree of FI_L3/4_ of PSM, and rFCSA_L4/5_ of the MFM. The AUC values generated by the nomogram were 0.711, 0.724, and 0.737 at 3, 12, and 24 months after the acute event, respectively. These values demonstrate that prediction accuracy was high, indicating that our nomogram will assist the selection of acute vertebral fracture treatment methods and prediction of vertebral fractures subsequent to OVCF.

OVCF prognosis is impacted by the high rate of subsequent vertebral fracture: approximately 22.5% of the patients in our study experienced subsequent vertebral fracture within 1 year, and 31.9% within 2 years. Mills et al. reported an incidence of subsequent vertebral fracture of 20.7% during 1-year follow-up [24]. Barton et al. demonstrated that the incidence of subsequent vertebral fracture within 2 years can be as high as 38% [10].Their results are broadly consistent with those of our study. The occurrence of subsequent vertebral fracture is affected by many factors. Of these, treatment methods and clinically related factors have been most extensively studied. With regard to treatment methods, vertebroplasty is a surgical treatment method widely used for clinical treatment of OVCF, and its association with subsequent vertebral fractures has been the subject of research. Multivariate Cox proportional hazards analysis confirmed that vertebroplasty was an independent risk factors for subsequent vertebral fracture in our study. Recent guidelines from the American Association of Clinical Endocrinologists [1] do not recommend vertebroplasty because Blasco et al. [25] and Bouza et al. [26] reported that vertebroplasty increases the risk of subsequent vertebral fracture, especially in adjacent vertebrae. A recent study also showed that subsequent vertebral fractures were more frequent, and occurred earlier, in the vertebroplasty group compared with the conservatively treated group [21]. These results are consistent with our own. The authors hypothesize two possible reasons. Firstly, the increased mobility of the patient after vertebroplasty, which increases the risk of subsequent vertebral fracture, and secondly, the possibility that the bone cement may leak into the intervertebral discs, resulting in uneven forces between the vertebrae, making the vertebrae more susceptible to fracture. However, a recent retrospective study of 36,135 patients found no difference in the incidence of subsequent vertebral fracture between patients who received vertebroplasty and those who received non-operative treatment, but anti-osteoporotic medications reduced the rate of subsequent vertebral fracture [24]. In addition, an earlier meta-analysis of 12 controlled clinical trials, randomized controlled trials, and prospective studies by Zhang et al. reached a similar conclusion that no increased risk of vertebral fracture was found after vertebroplasty compared with conservative treatment, especially in vertebrae adjacent to the treated vertebrae [27]. These apparently contradictory results may reflect inconsistent diagnostic evidence for subsequent vertebral fracture. Our diagnosis was based on MRI, whereas other studies only reviewed medical records as evidence of subsequent vertebral fracture. A review of the medical records to make a diagnosis of subsequent vertebral fracture would include some non-fracture cases because osteoporosis also results in loss of vertebral height, which may be misdiagnosed as a fracture.

With regard to clinically related factors, advanced age [14], low BMD [9, 17], and absence of anti-osteoporosis drugs [17, 24] may be associated with subsequent vertebral fracture. In this study, advanced age and low BMD were identified as risk factors by the univariate, but not the multivariate, Cox proportional hazards analysis. This result indicates that other factors play more important roles in the development of subsequent vertebral fractures, and in the process of compensating across risk factors. In this study, only 21.5% of the patients received regular anti-osteoporosis drug treatment. This factor was not different between the fracture and non-fracture groups, which is inconsistent with Mills et al. [24]. We attribute this apparent inconsistency to the small proportion of cases receiving anti-osteoporosis drug treatment in our study.

An important finding of our study was that rFCSA of the MFM at the L4/5 IVD level was an independent risk factor for subsequent vertebral fracture. Among the paraspinal muscles, the MFM is the strongest stabilizer of the lumbar spine [28]. The effect of the bilateral MFM accounts for more than two-thirds of the spine stiffness. This muscle group is able to generate large passive forces, and therefore provides passive resistance to lumbar flexion [29, 30]. Loss of rFCSA within the MFM disrupts the axial stabilization system, which in turn increases axial loading on the spine and relative motion between segments [30, 31]. Hence, we speculate that vertebral attachment to a dysfunctional MFM is less effective against perturbations and thus more susceptible to subsequent vertebral fracture. Consistent with this, previous research reported that incomplete MFM function was associated with bone nonunion after lumbar interbody fusion [22], new compression fracture after vertebroplasty [28], and OVCF [32]. We also found that, although the rFCSA and FI of the MFM at the L3/4 and L4/5 IVD levels were associated with fracture, only rFCSA of the MFM at the L4/5 IVD level was an independent risk factor, even for OVCF at other levels, indicating that this factor best reflects overall MFM function and its influence on subsequent vertebral fracture. This result is also similar to the finding that a low MFM is associated with two or more grades of disc degeneration [33] and increased risk of fall [34], and that pathological changes in the MF typically occur at the L4/5 IVD level [35]. It is consistent with Crawford et al., who reported that the degree of FI of MF at the L4/5 IVD level best represented the degree of FI of the MF in the entire lumbar region of [36]. From the above, it is clear that assessment of MF at the L4/5 IVD level in patients with acute OVCF may be predictive of subsequent vertebral fracture, which would help to identify more vulnerable and higher risk groups in clinical practice.

Another important finding of our study was that FI of the PSM at the L3/4 IVD level was an independent risk factor for subsequent vertebral fracture. The PSM, a core muscle closely related to walking and running, has emerged as a novel, validated surrogate marker for systemic skeletal muscle function [37, 38]. Increased fat infiltration in PSM may reduce functional muscle mass, leading to sarcopenia. Previous studies have also shown that changes in the PSM at the L3/4 IVD level are strongly associated with sarcopenia and significantly associated with many adverse outcomes [38–42]. Sarcopenia is associated with higher risk of falls and fractures [43], and is a major risk factor for loss of independence in older adults [44]. In addition, many studies reported that sarcopenia was associated with vertebral fracture [20, 32, 45–47]. The above results are consistent with those of this study.

This study had some limitations. First, it was a retrospective study for which some data could not be obtained or were incomplete. This limitation necessitated the exclusion of some patients, possibly introducing selection bias. Second, the FCSA and FI of the paraspinal muscles were evaluated from T2-weighted MRI data. In the future, we will prospectively adopt magnetic resonance water-fat separation technology for more accurate assessment of the paraspinal muscles. Third, we selectively included cases with new fractures during follow-up confirmed by MRI. Therefore, cases of new fractures without MR examination may have been excluded. Fourth, the use of disc area to adjust for the effect of body size on CSA may not be as authoritative as adjusting for height, but lumbar disc area correlates with height [48], and doing so is reasonable and obtainable on MRI images. Fifth, the study did not include all risk factors that may influence subsequent vertebral fractures, such as falls in the older adults, medications affecting BMD, and frailty.

In conclusion, our results suggest that PVP treatment, low rFCSA_L4/5_ of the MFM, and excessive FI_L3/4_ of PSM are independent risk factors for subsequent vertebral fracture. We integrated the above risk factors into a nomogram model, which could aid personalized assessment of recurrent fracture risks after acute vertebral fracture.

### Supplementary Information

Below is the link to the electronic supplementary material.

Supplementary file1 (DOCX 20 kb)

## Data Availability

The datasets generated or analyzed during the study are available from the corresponding author on reasonable request.

## Abbreviations

OVCF: Osteoporotic vertebral compression fracture
IVDs: Intervertebral discs
CSA: Cross-sectional area
FI: Fatty infiltration
BMD: Bone mineral density
MRI: Magnetic resonance imaging
PVP: Percutaneous vertebroplasty
