# Supplementary material for: Prediction of Subsequent Vertebral Fracture After Acute Osteoporotic Fractures from Clinical and Paraspinal Muscle Features
Source: Calcif Tissue Int. 2024 May 7;114(6):614–24. doi: 10.1007/s00223-024-01209-0 (PMC11090933; doi:10.1007/s00223-024-01209-0)
Supplement: Supplementary file 1 — Supplementary file1 (DOCX 20 kb) [file 223_2024_1209_MOESM1_ESM.docx]

**Supplementary material**

***Supplementary material 1. Imaging Procedures***

***Supplementary material 2. Percutaneous vertebroplasty***

***Supplementary material 3. Intraclass correlation coefficients (ICC) for inter- and intra-observer measurement reliability***

***1.Imaging Procedures***

**Lumbar MRI examinations**

MRI was performed using a 1.5-Tesla scanner (Magnetom Avanto; Siemens, Erlangen, Germany). Sagittal T1- and T2-weighted images, and axial T2-weighted images, were obtained. Individuals were placed in the supine position to keep the hips and knees slightly flexed, and to maintain a standardized lumbar position and symmetric alignment of the lower limbs.

Specific parameters of conventional MRI were as follows: **sagittal T1-weighted images**: pulse sequence, T1 fast spin-echo; repetition time (TR), 535 ms; echo time (TE), 11 ms; matrix, 320 × 256; echo train length, 3; field of view (FoV), 320 mm; number of excitations (NEX), 1; number of slice groups, 1; number of slices, 12; slice thickness, 4.0 mm; slice space, 0.4 mm; **sagittal T2-weighted images**: pulse sequence, T2 fast spin-echo; TR, 3,500 ms; TE, 110 ms; matrix, 320 × 256; echo train length, 3; FoV, 240 mm; NEX, 1; number of slice groups, 1; number of slices, 12; slice thickness, 4.0 mm; slice space, 0.4 mm; **axial T2-weighted images**: pulse sequence, T2 fast spin-echo; TR, 3,500 ms; TE, 108 ms; matrix, 384 × 288; echo train length, 3; FoV, 240 mm; NEX, 1; number of slice groups, 5; number of slices, 3; slice thickness, 4.0 mm; slice space, 0.4 mm. The conventional axial images included the intervertebral discs.

**Lumbar X-ray examinations**

Lumbar X-ray was performed using a digital radiography machine (Philips Medical Systems DMC GmbH, Hamburg, Germany) with parameters of 75 kV and 32 mAs for anteroposterior imaging, and 85 kVp and 32 mAs for lateral imaging.

***2.******Percutaneous vertebroplasty***

After local anesthesia (1% lidocaine), all surgical patients received bilateral or unilateral PVP in the prone position under the guidance of C-arm fluoroscopy. According to Jensen’s technique, under C-arm fluoroscopic control, 11- or 13-gauge bone biopsy needles were inserted into the pedicle, in a slightly descending trajectory or parallel to the superior and inferior edges of the pedicle. The needle was positioned optimally, as confirmed by C-arm fluoroscopy; the tip reached the anterior third of the vertebral body and midpoint of the midline. After the stylet was removed from the trocar, a polymethylmethacrylate mixture was instilled to fill the fractured bone. The cement injection process was performed slowly and strictly monitored under C-arm fluoroscopy in the lateral plane to avoid cement leakage. The bone cement filled the fractured vertebrae in the anterior third of the vertebral body as much as possible, thereby forming a mechanical column. The injection was immediately stopped if cement leakage was seen in the segmental vein, adjacent intervertebral disc, epidural space, or epidural vein. All patients were allowed to ambulate the day after percutaneous vertebroplasty surgery.

***3. Intraclass correlation coefficients (ICC) for inter- and intra-observer measurement reliability***

|  | Inter-observer ICC | *P* | Intra-observer ICC | *P* |
| --- | --- | --- | --- | --- |
| L3/4 IVD level |  |  |  |  |
| Area of R-PSM | 0.987 | 0.000 | 0.988 | 0.000 |
| Area of R-ESM | 0.971 | 0.000 | 0.977 | 0.000 |
| Area of R-MFM | 0.984 | 0.000 | 0.983 | 0.000 |
| Area of L-PSM | 0.984 | 0.000 | 0.989 | 0.000 |
| Area of L-ESM | 0.991 | 0.000 | 0.987 | 0.000 |
| Area of L-MFM | 0.981 | 0.000 | 0.973 | 0.000 |
| Area of L3/4 IVD | 0.972 | 0.000 | 0.965 | 0.000 |
| TCSA | 0.987 | 0.000 | 0.976 | 0.000 |
| L4/5 IVD level |  |  |  |  |
| Area of R-PSM | 0.990 | 0.000 | 0.989 | 0.000 |
| Area of R-ESM | 0.983 | 0.000 | 0.977 | 0.000 |
| Area of R-MFM | 0.977 | 0.000 | 0.984 | 0.000 |
| Area of L-PSM | 0.983 | 0.000 | 0.973 | 0.000 |
| Area of L-ESM | 0.985 | 0.000 | 0.979 | 0.000 |
| Area of L-MFM | 0.969 | 0.000 | 0.987 | 0.000 |
| Area of L4/5 IVD | 0.994 | 0.000 | 0.985 | 0.000 |
| TCSA | 0.986 | 0.000 | 0.985 | 0.000 |
